# Heart at Hand: The Role of Point-of-Care Cardiac Ultrasound in Internal Medicine

**DOI:** 10.3390/jcdd12100379

**Published:** 2025-09-24

**Authors:** Piero Tarantini, Francesco Cei, Fabiola Longhi, Aldo Fici, Salvatore Tupputi, Gino Solitro, Lucia Colavolpe, Stefania Marengo, Nicola Mumoli

**Affiliations:** 1 Department of Cardiovascular Medicine, Ospedale di Circolo, Via Arnaldo da Brescia, 1, 21052 Busto Arsizio, Italy; piero.tarantini@asst-valleolona.it (P.T.); aldo.fici@asst-valleolona.it (A.F.); salvatore.tupputi@asst-valleolona.it (S.T.); gino.solitro@asst-valleolona.it (G.S.); lucia.colavolpe@asst-valleolona.it (L.C.); 2 1st Internal Medicine, Department of Medicine, Azienda USL Toscana Centro, 50053 Empoli, Italy; francesco.cei90@gmail.com; 3 Department of Internal Medicine, AO Ordine Mauriziano, 10128 Turin, Italy; stefi.marengo@gmail.com

**Keywords:** echocardiography, ultrasound, bedside, internal medicine

## Abstract

Bedside echocardiography stands as a cornerstone diagnostic tool in internal medicine, offering rapid, real-time evaluation of cardiac structure and function across a wide spectrum of acute and chronic conditions. Its application, particularly when combined with lung and inferior vena cava (IVC) ultrasound, significantly enhances diagnostic accuracy for fluid balance assessment, dyspnea, and hypotensive states, guiding timely therapeutic decisions. Focused cardiac ultrasound (FoCUS) enables internists to assess left ventricular function, right atrial pressure, valvular abnormalities, and pericardial effusion, facilitating differentiation between cardiac and non-cardiac causes of symptoms such as dyspnea, chest pain, and hemodynamic instability. While operator-dependent, echocardiography can be effectively integrated into internal medicine practice through structured training programs that combine theoretical knowledge with supervised hands-on experience. This integration enhances clinical decision-making, optimizes patient management, and reduces the need for immediate specialist consultation. Widespread adoption of focused ultrasound techniques in internal medicine wards promises not only improved patient outcomes but also more efficient utilization of healthcare resources. Continued education and institutional support are fundamental to embedding echocardiography into routine care, ensuring internists are equipped to leverage this powerful bedside modality. This narrative review aims to underscore the transformative impact of bedside echocardiography in internal medicine, demonstrating its capacity, when combined with lung and IVC ultrasound, to optimize diagnostic pathways and treatment decisions across diverse acute and chronic settings.

## 1. Introduction

Within internal medicine, echocardiography stands as an indispensable diagnostic instrument, offering immediate and crucial insights into cardiac structure and function. Internal medicine wards manage a wide array of cardiovascular conditions, ranging from heart failure to acute chest syndromes, where rapid assessment of cardiac performance is critical not only for diagnosis but also for guiding management [1].

Heart failure remains the leading cause of hospitalization in internal medicine settings, making the assessment of left ventricular ejection fraction, diastolic function and chamber dimensions essential [2]. Echocardiography also aids in evaluating valvular function, wall motion abnormalities, and the characterization of cardiomyopathies [3].

Beyond chronic conditions, echocardiography plays a pivotal role in acute settings, assisting in managing patients with hypotension, dyspnea and chest pain, as well as diagnosing cardiac complications from infectious diseases, such as infective endocarditis. Bedside echocardiography thus enhances both diagnostic accuracy and therapeutic appropriateness in a wide range of clinical contexts [4].

This narrative review outlines the essential information derived from focused cardiac ultrasound in inpatient internal medicine, demonstrating its application in managing chronic conditions like heart failure and acute presentations such as dyspnea and hypotension.

## 2. Materials and Methods

This narrative review draws on a purposive sampling of MEDLINE-indexed literature (via PubMed) to provide a representative overview of the field, focusing on influential guidelines, recent clinical trials, and relevant narrative reviews published within the last decade. The objective was to identify key studies on the use of echocardiography and focused cardiac ultrasound (FoCUS) in internal medicine, particularly for the diagnosis and management of dyspnea, hypotension, heart failure, and chest pain.

Given the non-systematic design, the selection aimed to capture real-world applications and clinical outcomes of bedside echocardiography across diverse settings. Evidence on the diagnostic performance of FoCUS, as well as logistical and training challenges affecting its implementation in internal medicine wards, was also considered.

The search strategy included the following inclusion and exclusion criteria:

Inclusion criteria:

- Publication type: influential guidelines, clinical trials randomized controlled trials, observational studies, systematic reviews and meta-analysis, and narrative reviews (published in the last 10 years to avoid outdate data and represent the actual clinical practice)
- Subject matter: echocardiography and FoCUS in internal medicine.
- Clinical focus: diagnosis and management of dyspnea, hypotension, heart failure, and chest pain.
- Search terms: FoCUS, Bedside echocardiography, point of care cardiac echocardiography (POCUS), differential diagnosis of dyspnea, hypotension or shock, chest pain, heart failure
- Bibliographic review: inclusion of studies identified through reference lists.
- Practical relevance: preference for studies with direct applicability to real-world clinical practice and patient outcomes.

Exclusion criteria:

- Irrelevance to bedside practice: studies focusing exclusively on advanced or highly specialized echocardiographic techniques without applicability to internal medicine wards.
- Non-clinical content: technical or engineering papers without direct patient care implications.
- Obsolete evidence: publications based on outdated technology, protocols, or training models no longer in current use.
- Insufficient detail: articles lacking clear methodological description or outcome reporting, preventing meaningful clinical interpretation.
- Non-peer-reviewed sources: conference abstracts, opinion pieces, or educational materials not subjected to peer review.
- Population mismatch: studies conducted exclusively in pediatric, surgical, or highly selected outpatient cohorts not representative of internal medicine inpatients.

## 3. Learning Curve

The increasing availability of ultrasound machines in internal medicine wards has prompted the need for structured training in echocardiography [5]. However, ultrasound remains a highly operator-dependent tool, requiring both technical skill and clinical reasoning [6]. Many studies have demonstrated that non-cardiologists can acquire basic echocardiographic competencies with appropriate training [7,8].

**Figure 1:**
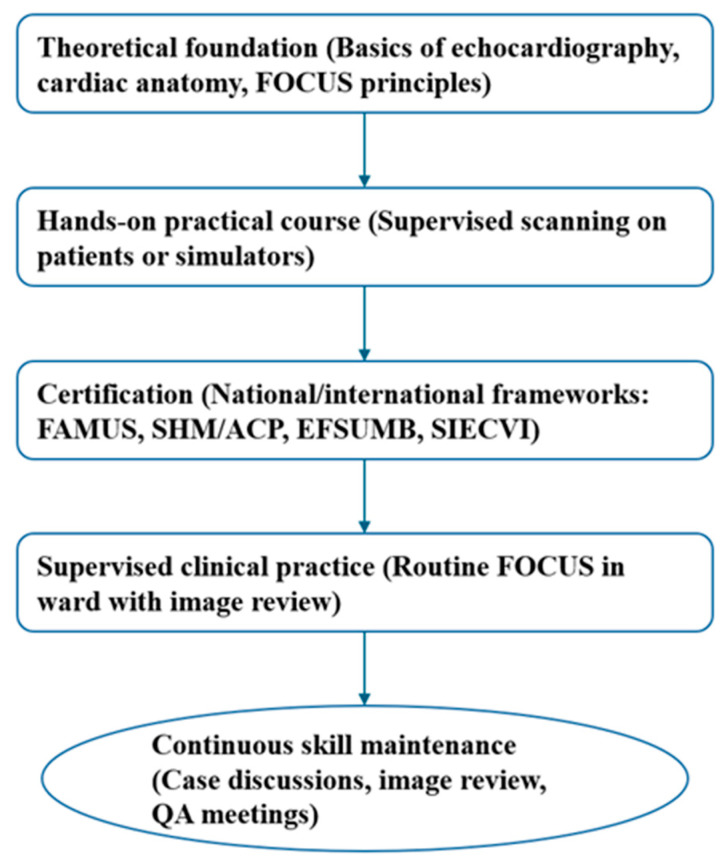
Proposed FOCUS training pathway for Internists.

Short courses, including both theoretical modules and hands-on practice, can enable internists to reliably and safely perform focused cardiac ultrasound exams [9]. It is therefore necessary to provide targeted training aimed at acquiring specific competencies, which must then be appropriately certified, as proposed by some of the main national and international frameworks for internists and non-cardiologist physicians, such as FAMUS in the UK, SHM/ACP in the US, EFSUMB in Europe and SIECVI in Italy. Moreover, in the era of artificial intelligence, powerful new tools are becoming part of the physician’s arsenal, such as simulators. A simulator-based curriculum improved medical student’s skills in an objective and quantifiable manner [10,11]. Self-directed learning with ultrasound simulators may be a scalable alternative to conventional supervised teaching with human models (Figure 1).

FoCUS is not a replacement for standard echocardiography but is a goal-directed, problem-oriented tool to answer specific clinical questions, such as:

- Is there left ventricular systolic dysfunction?
- Is the IVC dilated or collapsible?
- Is there pericardial effusion or tamponade?
- Are there signs of the right ventricular strain?

This simplified approach is particularly useful in emergency settings or during night shifts, when prompt decision-making is required [12]. Nonetheless, continuous supervision, feedback, and image review are essential to reinforce accuracy and avoid misinterpretation. Regular practice and case discussion are necessary to consolidate skills and ensure high-quality examinations [13].

## 4. Accuracy

Focused cardiac ultrasound provides reliable and accurate assessments of cardiac size, significant systolic and diastolic dysfunction, and pericardial effusion, with results comparable to those of standard echocardiography. This allows clinicians to rapidly obtain dependable information on the functionality and morphology of the heart and large vessels in patients admitted to medical departments [14]. These findings hold true even when the exam is performed by non-cardiologists or medical residents following a brief period of dedicated training, both with traditional and handheld ultrasound devices [15,16].

## 5. Echocardiography in Stable Hospitalized Patients

Bedside ultrasound, particularly focused on cardiac and pulmonary evaluation, plays a crucial role in internal medicine wards. Three common clinical scenarios benefit most from this approach, providing valuable diagnostic and therapeutic guidance: hospitalized patients presenting with symptoms suggestive of heart failure [17]; patients with newly detected cardiac murmurs [18]; and patients in whom infectious cardiac involvement must be excluded, such as in fever of unknown origin or bacteremia [19]. These scenarios are analyzed below.

**a.**  **Evaluation of suspected heart failure**

Patients admitted to internal medicine wards frequently present with symptoms compatible with heart failure, such as peripheral edema, dyspnea, persistent cough, and marked fatigue. However, these findings lack specificity and may also result from non-cardiac conditions, including hypoalbuminemia, chronic venous insufficiency, respiratory disease, or anemia. A third heart sound has sensitivity of approximately 13% and specificity of 99%; jugular venous distension a sensibility of 39% and a specificity of 92%; pulmonary rales of 60% and of 78%; and peripheral edema of 50% and of 78%, respectively—figures that contextualize the added value of FoCUS in refining bedside probability estimates [20]. While clinical evaluation remains fundamental, bedside ultrasonography, especially focused cardiac ultrasound, provides immediate and relevant information on cardiac morphology and function as well as systemic congestion [21]. This aids differentiation between cardiogenic and non-cardiogenic causes. A structured three-step approach is proposed for rapid evaluation of suspected heart failure [22]. It is important to note that FoCUS and lung ultrasound are distinct ultrasound applications that should be combined for the bedside assessment of heart failure. Evaluation of the IVC is a standard component of FoCUS and should not be performed in isolation without a comprehensive cardiac assessment.

*1.*  *Focused Cardiac Ultrasound*

FoCUS refers to a point-of-care cardiac ultrasound performed using a standardized, yet limited, scanning protocol as an extension of the clinical examination [23]. It is carried out by an operator who may not be fully trained in comprehensive echocardiography but has received appropriate training in FoCUS. This individual is typically responsible for immediate decision-making and/or treatment. Unlike traditional comprehensive echocardiographic studies, which can be time-consuming and require specialized training, FoCUS allows for rapid and effective assessment of both left and right ventricular function, as well as the detection of pericardial or pleural effusions.

In the acute setting, FoCUS facilitates the quick identification of life-threatening abnormalities, enabling clinicians to initiate therapy without delay. The technique uses binary questioning principles (e.g., is the left ventricle functioning normally? Is there evidence of pericardial effusion?), which aids in swift decision-making. The preliminary results obtained through FoCUS guide immediate interventions while providing a foundation for further detailed studies. A follow-up, advanced echocardiographic evaluation refines the initial diagnosis, providing a more tailored assessment of hemodynamic status. This secondary evaluation can assess cardiac responses, such as in cases of vasoplegia associated with septic shock, or determine the need for interventions targeting the right or left heart, intravascular volume, or pericardial effusions. No other bedside diagnostic tool matches the speed and diagnostic specificity of focused echocardiography, making it an indispensable part of the acute care clinician’s toolkit [24,25].

It is imperative that FoCUS is never misrepresented as a comprehensive echocardiographic examination and should only be performed by operators who have completed the appropriate education and training programs, and who have a full understanding of the scope and limitations of this diagnostic tool. In cases where the information obtained from a FoCUS examination is insufficient to guide immediate or definitive patient care, the patient must be referred for a comprehensive echocardiographic examination as soon as possible, in alignment with clinical priorities [26].

**Figure 2:**
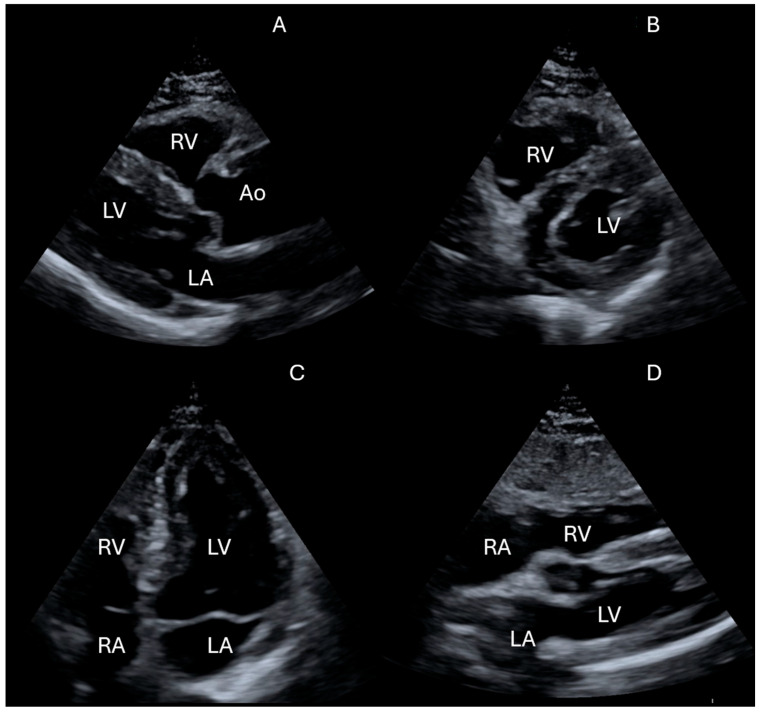
Basic FoCUS views commonly used in bedside echocardiographic assessment. (**A**) Parasternal long-axis. (**B**) Parasternal short-axis. (**C**) Apical 4-chamber. (**D**) Subcostal 4-chamber. (RV) Right ventricle. (LV) Left ventricle. (RA) Right atrium. (LA) Left atrium. (Ao) Aorta.

The main modalities utilized in FoCUS include 2D imaging, M-mode, and Color Doppler. The key acoustic windows for cardiac ultrasound include the following (Figure 2).

The parasternal long axis view (PLAX) allows for the evaluation of ventricular hypertrophy and pathology of the aortic and mitral valves, while the parasternal short axis view (PSAX) is useful for assessing pulmonary artery systolic pressure, aortic valve morphology, regional wall motion abnormalities of the left ventricle, and D-shaped septal flattening. The apical 4-chamber view (A4CH) provides a comprehensive assessment of left ventricular ejection fraction, diastolic function, right ventricular systolic function, and chamber sizes.

**Figure 3:**
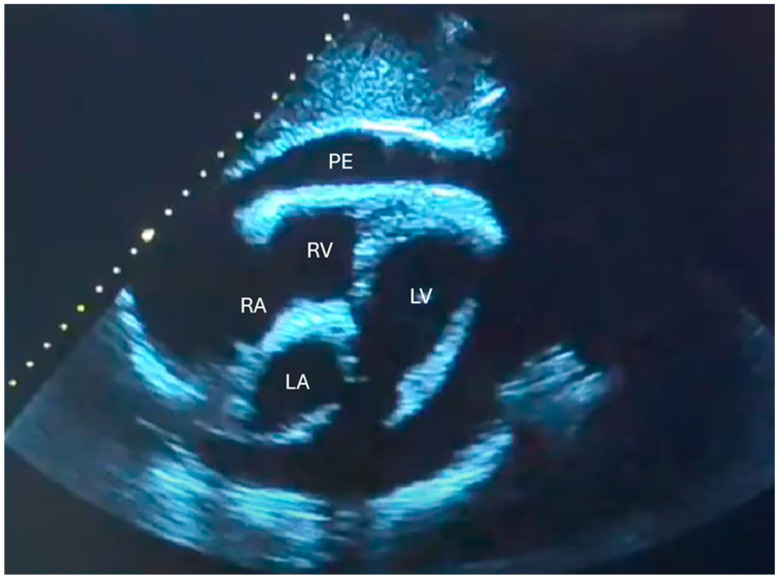
Pericardial effusion (PE). (RV) Right ventricle. (LV) Left ventricle. (RA) Right atrium. (LA) Left atrium.

Finally, the subcostal 4-chamber view (S4CH) is especially indicated in elderly or bedridden patients, as it is well-suited for detecting pericardial effusion (Figure 3) and atrial septal defects. Even when image quality is suboptimal, obtaining data from at least one acoustic window significantly supports diagnosis. Integrating cardiac, lung, and IVC ultrasound findings enables a comprehensive clinical evaluation and guides optimal management.

*2.*  *Assessment of right atrial pressure*

As part of FoCUS, the assessment of volume status is an important component in the evaluation of cardiac patients. Estimation of right atrial pressure (RAP) through measurement of the IVC diameter and its collapsibility during a sniff maneuver is widely endorsed by current echocardiographic guidelines [27]. A normal RAP is suggested when the IVC diameter is less than or equal to 2.1 cm, with greater than 50% inspiratory collapse, which is consistent with a low RAP of 0–5 mmHg. In such cases, cardiogenic dyspnea is less likely. Conversely, an elevated RAP is indicated by an IVC diameter greater than 2.1 cm and less than 50% collapse, which points to an RAP in the range of 10–20 mmHg, often reflecting volume overload. An intermediate RAP, approximately 8 mmHg, can be inferred in cases where IVC collapse and diameter fall between these two extremes (Table 1). Accurate interpretation of RAP estimation requires integration with clinical history, physical examination, and additional diagnostic tools, such as laboratory tests. It is important to recognize that RAP is a complex parameter influenced by volume status, systemic and pulmonary vascular resistances, intrathoracic and intrapericardial pressures, and atrial compliance. Therefore, it does not directly represent the patient’s total blood volume and is affected by multiple non-volume-related factors [28]. For example, in mechanically ventilated patients, IVC parameters show poor correlation with RAP. Intense athletic training has also been shown to cause IVC dilation without accompanying increases in RAP. Additionally, patients with a history of vasovagal syncope tend to have larger IVC diameters. Any obstruction in the vena cava, such as abnormalities of the Eustachian valve or narrowing at the junction between the IVC and the right atrium, can also lead to the IVC being an unreliable surrogate for right atrial pressure. Severe tricuspid valve regurgitation may further cause significant variability in IVC parameters, resulting in fluctuating and potentially inaccurate findings. Finally, conditions that lead to liver tissue stiffening, such as cirrhosis or liver fibrosis, can also impact on the diameter and collapsibility of the hepatic segment of the IVC, limiting the utility of IVC measurements in these patients [29].

*3.*  *Lung ultrasound evaluation*

**Figure 4:**
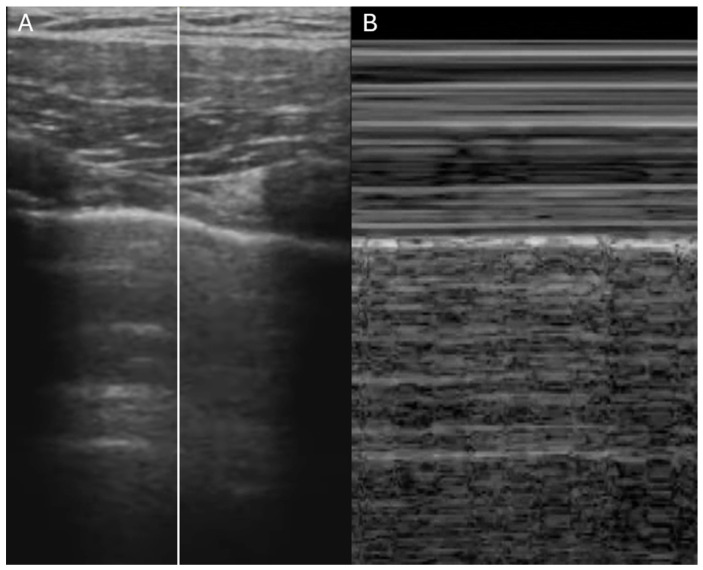
Lung ultrasound showing normal pleural sliding. (**A**) B-mode. (**B**) M-mode showing “seashore sign”.

**Figure 5:**
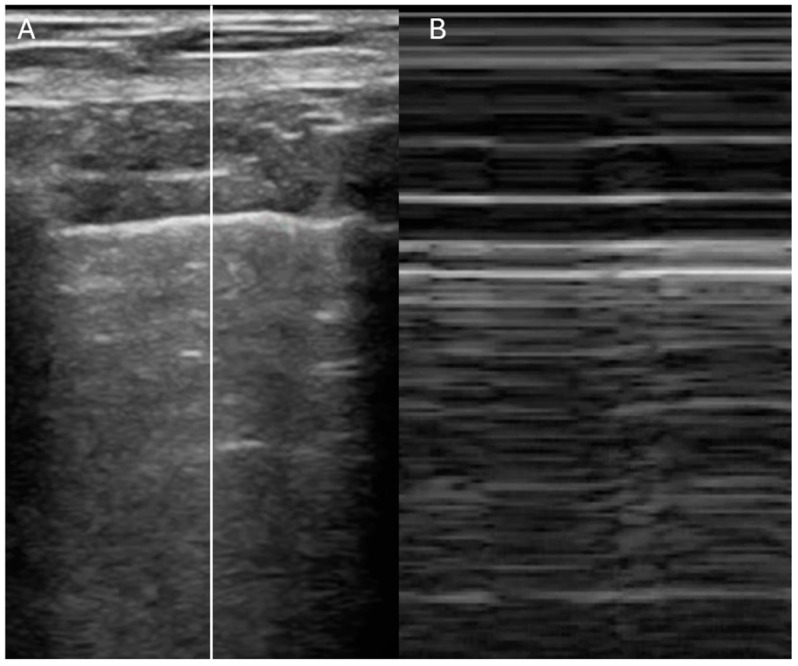
Lung ultrasound showing absent pleural sliding. (**A**) B-mode. (**B**) M-mode showing “barcode sign”.

**Figure 6:**
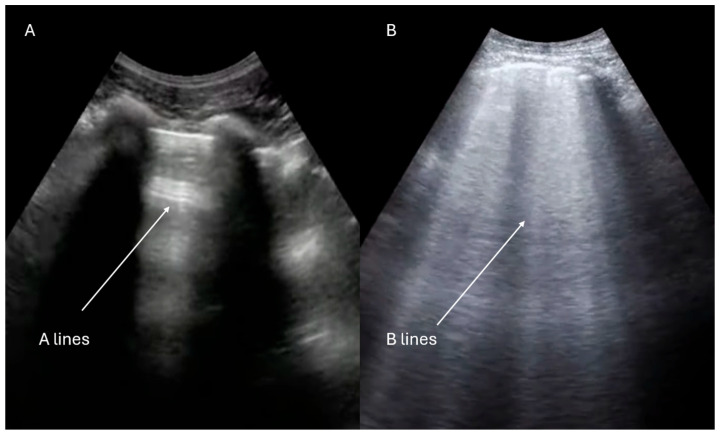
Lung ultrasound in B-mode. (**A**) A-pattern, characterized by horizontal A-lines. (**B**) B-pattern, characterized by vertical B-lines.

Lung ultrasound is a rapid, radiation-free diagnostic tool that is increasingly recognized as a first-line approach in the evaluation of dyspneic patients [30,31]. The initial assessment begins with the evaluation of pleural sliding using a high-frequency linear probe. The presence of the “seashore sign” (Figure 4) on M-mode imaging (above the pleural line the pattern is stratified due to motionless of soft tissues, whereas below the pleural line the pattern is sandy) indicates that the lung moves at the chest wall and so it is considered a reliable indicator that rules out pneumothorax, whereas the presence of the “barcode sign” (Figure 5) (standardized stratified pattern below and above the pleural line), indicates total absence of pleural motion, thus serving as an ultrasound sign of pneumothorax [32]. Following this, the lung parenchyma is examined for pleural effusions, consolidations, or artifacts that can provide insights into the degree of lung aeration. Three primary ultrasound artifacts patterns are typically observed during this evaluation (Figure 6). The A-pattern, characterized by repetition of horizontal A-lines that run parallel to the pleural line, suggests that the lung is normally aerated. The B-pattern, which is defined by the presence of at least three vertical B-lines per field, indicates interstitial or alveolar involvement. This pattern is commonly seen in conditions such as pulmonary edema, but it can also be present in interstitial pneumonia or pulmonary fibrosis. Finally, the white lung pattern, marked by the coalescence of B-lines, is indicative of severe interstitial or alveolar congestion, reflecting a more critical level of lung involvement [33].

**b.**  **Assessment of new cardiac murmurs**

Cardiac murmurs are a common finding in clinical practice, especially in internal medicine, and are frequently encountered during routine physical examinations. These abnormal heart sounds can be indicative of a range of cardiac conditions, from benign to life-threatening. However, auscultation alone often lacks the specificity needed to accurately determine the origin, severity, or clinical significance of a murmur. In many cases, murmurs are functional or innocent, typically observed in young individuals or those with no underlying heart disease, and do not carry pathological significance [34]. Such benign murmurs may arise from increased blood flow due to fever, anemia, or physical activity, and generally do not require further investigation.

On the other hand, significant valvular diseases, such as mitral regurgitation, aortic stenosis, or infective endocarditis, may present with faint or subtle murmurs that can easily be overlooked during auscultation. For example, in suspected aortic stenosis, meta-analytic data show that a diminished second heart sound (S2) is moderately sensitive and highly specific (sensitivity 0.59, specificity 0.95), and a delayed carotid upstroke performs similarly (0.57, 0.94), whereas a systolic murmur radiating to the neck is highly sensitive (0.93) but less specific (0.66) [35]. In these cases, the absence of a prominent murmur does not rule out the presence of severe underlying pathology. Therefore, a thorough assessment of a new murmur requires careful consideration of clinical context, patient history, and the use of supplementary diagnostic tools. Focused echocardiography has become an indispensable tool for the evaluation of new cardiac murmurs. The integration of physical examination and ultrasound facilitates accurate diagnosis, prompt recognition of serious cardiac conditions, and optimized therapeutic decisions.

The first step in the diagnostic approach to a heart murmur using Focused Cardiac Ultrasound is to identify the compromised valve [36]. For this purpose, the use of 2D echocardiography and Color Doppler imaging is essential. In cases of valvular insufficiency, identification is relatively straightforward, as an abnormal flow, referred to as a “regurgitant jet,” is observed, directed opposite to the normal physiological flow. For example, in aortic insufficiency, the regurgitant blood flow is directed toward the left ventricle during diastole. Conversely, in mitral insufficiency, regurgitation is observed into the left atrium during systole.

Valvular stenosis, on the other hand, presents a more complex diagnostic challenge. In these cases, a detailed analysis of the valve morphology and the motion of the valve leaflets is of paramount importance. Stenotic valves often show signs of calcification and limited leaflet mobility, which must be carefully evaluated [37]. Once the valvular defect has been identified, further assessment with a comprehensive echocardiographic examination can be carried out to determine the severity of the valvulopathy.

FoCUS not only enables the identification of valvular pathology but also provides insights into potential underlying causes (e.g., leaflet flail, prolapse, valvular tethering, endocarditic vegetation), associated complications (such as left ventricular hypertrophy in the context of aortic stenosis and left atrial dilation in the setting of mitral insufficiency), or hemodynamic implications (e.g., peripheral venous stasis in the case of severe tricuspid insufficiency or pulmonary congestion in severe mitral valve disease).

**c.**  **Assessment of suspected infective endocarditis**

In internal medicine wards, it is common to encounter clinical situations where it is necessary to exclude a cardiac infectious involvement, such as in patients with bacteremia or fever of unknown origin. Infective endocarditis (IE), although relatively rare, is a severe condition with rapid progression and high mortality if it is not treated in a timely fashion.

Traditional physical examination has very limited sensitivity and specificity when diagnosing bacterial endocarditis. Focused cardiac ultrasound can assist with the diagnosis by evaluating for valvular regurgitation or visible vegetation, and reduces dependency on immediate specialist consultation, critical in high-acuity settings [38].

Echocardiographic detection of echogenic masses attached to valvular structures in patients with clinical suspicion of IE, especially if associated with new valvular regurgitation, raises suspicion of vegetations. Transesophageal echocardiography (TEE) is frequently necessary for detailed morphological and functional lesion characterization. Importantly, a negative transthoracic echocardiography does not exclude IE when clinical probability is high; TEE remains the gold standard follow-up examination [39,40].

Another role of FoCUS in the context of endocarditis is its use in monitoring over time and detecting potential failure of antibiotic therapy. For instance, it can help identify an increase in vegetation size or the development of complications such as abscess formation or mechanical valve damage, including chordal rupture or leaflet perforation [41]. The ability to perform a bedside echocardiographic exam allows for close monitoring of the patient without the need for continuous consultation with other specialists, which is often impractical in clinical routine.

Early incorporation of bedside echocardiography in the diagnostic and therapeutic pathway for suspected IE enables timely, targeted, and potentially life-saving intervention.

## 6. Echocardiography in Internal Medicine Emergencies

As in routine clinical practice, bedside ultrasound, particularly transthoracic echocardiography, plays a crucial role in emergency situations that frequently complicate the hospitalization of patients in Internal Medicine wards, even in the identification of reversible causes of arrest in the critically ill, such as pulmonary embolism and cardiac tamponade, allowing early medical treatment and early transfer to the operating room for definitive surgical treatment [42]. It serves as an essential tool to rapidly guide the diagnostic process and optimize therapeutic strategies [43,44].

Unlike the Emergency Department setting, where acutely symptomatic patients are typically assessed in a multidisciplinary manner and have prompt access to laboratory and radiologic diagnostics, acute clinical deterioration in the ward often requires rapid assessment using locally available resources. In this context, the ability to perform echocardiography directly at the patient’s bedside proves extremely valuable, facilitating clinical decision-making and improving overall management. Moreover, in internal medicine wards, the use of focused cardiac ultrasound has been shown to play not only a diagnostic but also a therapeutic role [45].

The following sections will examine the main emergency clinical scenarios (acute dyspnea, hemodynamic instability and chest pain) in which echocardiography can significantly impact diagnosis, treatment, and prognosis.

**a.**  **Acute dyspnea**

**Figure 7:**
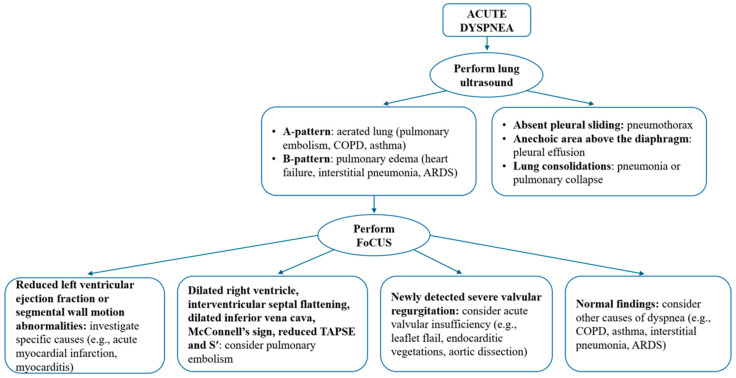
Approach protocol for the patient with acute dyspnea proposed by the authors.

The primary approach to a hospitalized patient who develops sudden dyspnea is to differentiate between cardiac and non-cardiac causes. As previously discussed, the initial evaluation of a patient with dyspnea typically includes lung ultrasound [46,47]. After excluding severe conditions such as pneumothorax, massive pleural effusion, and/or large consolidations, the next step is to conduct a cardiac evaluation (Figure 7). In the acute care setting, several key echocardiographic parameters warrant careful consideration. Assessment of left ventricular systolic function is paramount. Findings such as reduced left ventricular ejection fraction, or segmental wall motion abnormalities (hypokinesia or akinesia) may indicate acute heart failure, potentially linked to acute or chronic ischemic heart disease, myocarditis, Takotsubo syndrome, or other etiologies. Furthermore, left ventricular complications of ischemia, such as aneurysmal dilation or free wall rupture, may also be observed. Concurrently, it is crucial to evaluate the right ventricle for abnormalities indicative of acute cardiac compromise, such as those seen in the context of pulmonary embolism (further discussed in the subsequent section) [48]. Particular attention should also be directed towards the structure and function of cardiac valves. The presence of severe valvular abnormalities, especially acute regurgitation resulting from conditions like ruptured chordae tendineae or papillary muscles, acute aortic dissection involving the valvular apparatus, or valvular perforations secondary to endocarditis, can manifest as acute dyspnea.

**b.**  **Hemodynamic instability**

**Figure 8:**
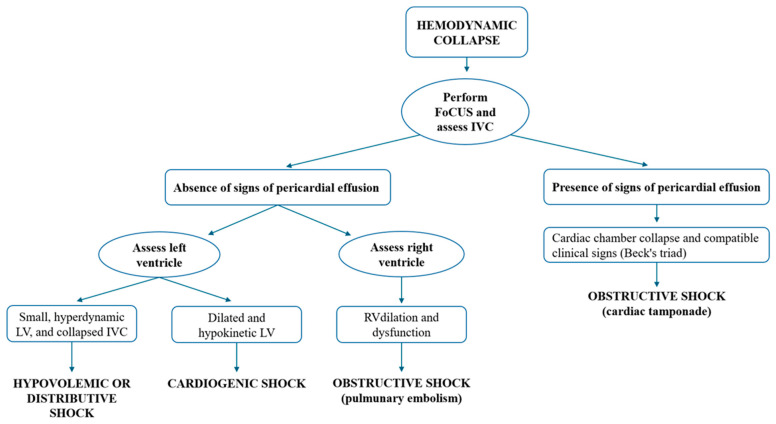
Approach protocol for the patient with hemodynamic instability proposed by the authors.

In patients presenting with shock or hemodynamic collapse, an initial echocardiographic evaluation is crucial for determining the underlying cause (Figure 8). The assessment begins with evaluating left ventricular morphology and function. A small, hyperdynamic LV, accompanied by a collapsed inferior vena cava, suggests hypovolemia. In contrast, a dilated and hypokinetic LV may indicate acute or acute-on-chronic heart failure, commonly observed in conditions such as myocardial infarction, myocarditis, or tachycardia-induced cardiomyopathy [49]. Right ventricular (RV) dilation and dysfunction are also key findings in such cases. Acute RV dysfunction, particularly when accompanied by signs of deep vein thrombosis or abnormal findings on lower limb venous Doppler ultrasound, should raise suspicion of pulmonary embolism. RV dilation is defined by a basal diameter exceeding 42 mm or an RV/LV basal ratio greater than 1.0. RV systolic function can be assessed by measuring Tricuspid Annular Plane Systolic Excursion (TAPSE), where values below 16 mm are considered pathological.

In cases of acute pulmonary embolism, additional echocardiographic findings may include preserved apical contractility, referred to as McConnell’s sign and flattening of the interventricular septum (the D-shape of the LV) [50,51]. In some cases, a mobile thrombus may also be visualized within the right heart chambers. Lastly, echocardiography serves as the first-line diagnostic tool in suspected cardiac tamponade. The subcostal window is considered the most reliable approach for detecting pericardial effusions, as the most dependent area of the pericardium lies closest to the probe’s face [52]. Pericardial effusions can be classified as “small,” “moderate,” or “large.” This classification is typically qualitative, though the effusion can be quantitatively assessed by measuring the largest fluid pocket at end-diastole, perpendicular to the heart’s surface. According to convention, small effusions are those less than 1 cm, moderate effusions range from 1 to 2 cm, and large effusions exceed 2 cm [53]. While moderate to large effusions are more likely to impact hemodynamics, even small effusions can lead to tamponade physiology. The identification of cardiac chamber collapse (collapse of the right atrium, collapse of the right ventricle, and finally LV collapse) along with, a plethoric IVC, and a compatible clinical presentation (such as hypotension, jugular venous distension, and muffled heart sounds), strongly suggests cardiac tamponade.

**c.**  **Acute chest pain**

In the setting of acute chest pain, echocardiography plays a pivotal role in the rapid assessment of the most common and potentially life-threatening cardiac causes [54]. In cases of acute coronary syndrome, transthoracic echocardiography allows for the early detection of regional wall motion abnormalities, such as hypokinesia, akinesia, or dyskinesia. This facilitates an early diagnosis, particularly in atypical or silent presentations, such as in elderly or diabetic patients, and supports prompt referral to the catheterization laboratory, thus optimizing time-sensitive management (time is myocardium). Furthermore, echocardiography is crucial for identifying rare but severe mechanical complications of myocardial infarction, including interventricular septal rupture with left-to-right shunting, papillary muscle rupture leading to acute mitral regurgitation, and the formation of left ventricular aneurysms or pseudoaneurysms, conditions that require urgent surgical intervention. Another critical diagnosis is acute aortic dissection, which can be detected by TTE when the intimal flap involves the proximal ascending aorta or aortic arch (in the latter case, evaluation can be performed via the suprasternal window) [55]. The presence of an intimal flap is highly specific but not sensitive for dissection [56]. This condition may also be associated with dilation of the aortic root or ascending aorta, acute aortic regurgitation and pericardial effusion.

Additional potentially life-threatening conditions that can be rapidly identified through echocardiography include pulmonary embolism, acute myopericarditis, acute valvular disease, and cardiac tamponade, all of which demand timely and accurate diagnostic evaluation and management [57].

## 7. Limitations

Despite the advantages of implementing FoCUS in internal medicine wards, several limitations and potential pitfalls need to be considered.

One major challenge is the lack of universal training standards. Currently, FoCUS training programs vary widely across countries and institutions, with heterogeneous curricula and certification processes (e.g., FAMUS, SHM/ACP, EFSUMB, SIECVI). This fragmentation generates disparities in operator competence and may compromise diagnostic accuracy. For this reason, structured training pathways are essential, based on shared minimum competencies and progressive clinical supervision [58,59].

Another concern is the risk of overconfidence and uncontrolled scope expansion. The immediate availability of echocardiographic images may lead less experienced internists to overestimate the reliability of their findings, resulting in misdiagnosis, delays in referral to specialists, or underuse of alternative diagnostic modalities. Evidence shows that interpretative errors are a real risk, particularly among less experienced physicians, in complex cases, or in patients with atypical echocardiographic phenotypes. It is important to emphasize that FoCUS was conceived as a goal-directed technique, designed to address specific binary clinical questions (e.g., left ventricular function, pericardial effusion, acute valvular dysfunction). Its inappropriate use as a surrogate for a comprehensive echocardiographic study may mislead clinical decision-making and lower the quality of care [60].

To mitigate these risks, the following measures are recommended:

- Competency standards and accreditation: structured training programs with objective skills assessment and stepwise certification.
- Image archiving: digital storage of echocardiographic clips enables periodic audits, remote supervision, and consultation with specialists, ensuring traceability.
- Continuing education: regular refresher courses and retraining are crucial to maintain competencies and prevent quality drift.
- Audit and peer review: periodic case discussions and image reviews serve as key tools for continuous quality improvement.
- The report should be clear and transparent: the clinician must always specify the scope and depth of the examination performed and state any technical limitations. This prevents ambiguity and makes the interpretive boundaries of the findings explicit to both patients and colleagues.

To help clear report, Table 2 differentiating between basic FoCUS, advanced FoCUS and complete echocardiography.

Beyond training and interpretation issues, equipment availability also represents a barrier to the broader adoption of FoCUS. The recent introduction of handheld ultrasound devices has improved accessibility and has proven reliable for several applications, such as the assessment of cardiac chamber morphology and function, inferior vena cava evaluation, and basic lung ultrasound (pleural effusion, B-lines), particularly when performed by experienced physicians [61,62]. A future goal will be to further enhance the accuracy and performance of portable devices, thereby increasing the reliability of bedside ultrasound and facilitating its integration into daily clinical practice.

## 8. Conclusions

Bedside echocardiography is transforming internal medicine practice, offering real-time, actionable insights in both acute and chronic care settings. When performed by trained internal medicine physician, focused cardiac ultrasound is a safe, efficient, and cost-effective tool that enhances clinical decision-making. Its integration with lung and IVC ultrasound provides a comprehensive physiological assessment that is particularly valuable in patients with dyspnea, heart failure, or hemodynamic instability. Given its high diagnostic yield in common clinical syndromes, wider adoption of FoCUS by internists is essential for improving patient outcomes, reducing reliance on specialist consultations, and optimizing resource utilization.

To realize the full potential of ultrasound in internal medicine, structured and widely accessible training pathways are essential. Competency-based programs, continuous mentorship, and institutional support are key to embedding echocardiography into everyday clinical workflows.

The heart is truly at hand, provided we are equipped with the skills to visualize it.

## Figures and Tables

**Table 1:** Estimation of RA pressure based on IVC diameter and collapse.

IVC Diameter	Collapse with Sniff	Estimated RA Pressure
≤2.1 cm	>50%	0–5 mmHg
≤2.1 cm	<50%	5–10 mmHg
>2.1 cm	<50%	10–20 mmHg

**Table 2:** FoCUS vs. Advanced FoCUS vs. Comprehensive Echocardiography (TTE)—Parameters and Measurements.

Parameter/Measurement	Basic Cardiac FoCUS/PoCUS	Advanced FoCUS	Comprehensive Echocardiography (TTE)
LV systolic function (visual)	Yes (qualitative only)	Yes (semi-quantitative; visual + basic metrics)	Yes (quantitative, biplane Simpson/3D)
Regional wall-motion abnormalities	Limited (gross only)	Yes (screening)	Yes (standard)
LV ejection fraction (numeric)	No	Limited (single-plane or eyeball estimate)	Yes (Simpson/3D)
RV size (base/mid/RA)	Yes (screening)	Yes (with basic metrics)	Yes (standard)
RV function (TAPSE/S’/FAC)	No	Yes (TAPSE/S’ optional)	Yes
IVC diameter & collapsibility	Yes	Yes (with trending)	Yes (plus RA pressure estimate)
Pericardial effusion—detection	Yes	Yes	Yes
Tamponade physiology (RV diastolic collapse, inflow variation)	Limited (signs only)	Yes (qualitative + basic Doppler optional)	Yes (full Doppler assessment)
Valvular disease—screening	Limited (gross lesions/murmur correlation)	Yes (screening + semi-quant)	Yes (comprehensive quantification)
Valvular quantification (continuity equation, PHT, vena contracta, PISA)	No	No	Yes
Diastolic function (E/A, e’, E/e’, LA vol, TRv)	No	Limited (E/A or E/e’ if available)	Yes (multi-parameter)
Pulmonary pressures (PASP/mean PAP)	No	Limited (TRv if feasible)	Yes (standard when feasible)
Cardiac output/stroke volume (LVOT VTI)	No	Limited (trend with VTI)	Yes (quantitative)
Aortic root/ascending aorta (screening)	Limited (root only)	Yes (screening)	Yes (standard + dimensions)
Aortic stenosis severity (continuity equation)	No	Limited (peak velocity if Doppler available)	Yes (Vmax, mean gradient, AVA)
Endocarditis features (vegetation/abscess)	No	Limited (large vegetations only)	Yes (TTE; TEE often required)
Intracardiac thrombus/masses	Limited (apical thrombus if obvious)	Limited (screening)	Yes (contrast/TEE/CMR as needed)
Lung ultrasound adjunct (B-lines, effusions, PTX signs)	Yes (integrated FoCUS)	Yes (integrated FoCUS)	Optional adjunct
Procedure guidance (pericardiocentesis, lines)	Yes (basic guidance)	Yes (enhanced guidance)	Yes (usually not required for full TTE)
Serial monitoring on ward/ED/ICU	Yes (rapid trending)	Yes (semi-quant trending)	Limited (resource-intensive)

Notes: “Yes/No/Limited” denote expected capability in typical practice; operator experience and equipment may expand or restrict capabilities. Abbreviations: LV/RV = left/right ventricle; RA = right atrium; TAPSE = tricuspid annular plane systolic excursion; S’ = tissue Doppler systolic velocity; FAC = fractional area change; IVC = inferior vena cava; PHT = pressure half-time; PISA = proximal isovelocity surface area; LA = left atrium; TRv = tricuspid regurgitation velocity; PASP = pulmonary artery systolic pressure; LVOT = left ventricular outflow tract; VTI = velocity–time integral; Vmax = peak velocity; AVA = aortic valve area; TTE/TEE = transthoracic/transesophageal echocardiography; GLS = global longitudinal strain; CMR = cardiac magnetic resonance; PTX = pneumothorax; PoCUS = point-of-care ultrasound.

## Data Availability

The original contributions presented in this study are included in the article. Further inquiries can be directed to the corresponding author.

## Abbreviations

The following abbreviations are used in this manuscript:

IVC	Inferior vena cava
FoCUS	Focused cardiac ultrasound
RAP	Right atrial pressure
IE	Infective endocarditis
TEE	Transesophageal echocardiography

## References

[1] Johri A.M., Glass C., Hill B., Jensen T., Puentes W., Olusanya O., Capizzano J.N., Dancel R., Reierson K., Reisinger N. (2023). The Evolution of Cardiovascular Ultrasound: A Review of Cardiac Point-of-Care Ultrasound (POCUS) Across Specialties. Am. J. Med..

[2] Kellett J., Deane B. (2007). The diagnoses and co-morbidity encountered in the hospital practice of acute internal medicine. Eur. J. Intern. Med..

[3] Trasca L., Popescu M.R., Popescu A.C., Balanescu S.M. (2022). Echocardiography in the Diagnosis of Cardiomyopathies: Current Status and Future Directions. Rev. Cardiovasc. Med..

[4] Cogliati C., Torzillo D., Casella F., Del Medico M., Montano N. (2016). Bedside echocardiography in internal medicine: Which are the key questions and answers for our decision-making?. Ital. J. Med..

[5] Topoll A., Berlacher K. (2022). Enhancing Echocardiography Education in Fellowship. J. Am. Soc. Echocardiogr..

[6] Mumoli N., Marengo S. (2024). Clinical utility of echocardiography in internal medicine: A narrative review. Ital. J. Med..

[7] Kühl M., Wagner R., Bauder M., Fenik Y., Riessen R., Lammerding-Köppel M., Gawaz M., Fateh-Moghadam S., Weyrich P., Celebi N. (2012). Student tutors for hands-on training in focused emergency echocardiography--a randomized controlled trial. BMC Med. Educ..

[8] Kim K.H., Jung J.Y., Park J.W., Lee M.S., Lee Y.H. (2021). Operating bedside cardiac ultrasound program in emergency medicine residency: A retrospective observation study from the perspective of performance improvement. PLoS ONE.

[9] Westwood M., Almeida A.G., Barbato E., Delgado V., Dellegrottaglie S., Fox K.F., Gargani L., Huber K., Maurovich-Horvat P., Merino J.L. (2023). Competency-based cardiac imaging for patient-centred care. A statement of the European Society of Cardiology (ESC). With the contribution of the European Association of Cardiovascular Imaging (EACVI), and the support of the Association of Cardiovascular Nursing & Allied Professions (ACNAP), the Association for Acute CardioVascular Care (ACVC), the European Association of Preventive Cardiology (EAPC), the European Association of Percutaneous Cardiovascular Interventions (EAPCI), the European Heart Rhythm Association (EHRA), and the Heart Failure Association (HFA) of the ESC. Eur. Heart J. Imaging Methods Pract..

[10] Elison D.M., McConnaughey S., Freeman R.V., Sheehan F.H. (2020). Focused cardiac ultrasound training in medical students: Using an independent, simulator-based curriculum to objectively measure skill acquisition and learning curve. Echocardiography.

[11] Canty D., Barth J., Yang Y., Peters N., Palmer A., Royse A., Royse C. (2019). Comparison of learning outcomes for teaching focused cardiac ultrasound to physicians: A supervised human model course versus an eLearning guided self- directed simulator course. J. Crit. Care.

[12] Labovitz A.J., Noble V.E., Bierig M., Goldstein S.A., Jones R., Kort S., Porter T.R., Spencer K.T., Tayal V.S., Wei K. (2010). Focused cardiac ultrasound in the emergent setting: A consensus statement of the American Society of Echocardiography and American College of Emergency Physicians. J. Am. Soc. Echocardiogr..

[13] Kimura B.J. (2017). Point-of-care cardiac ultrasound techniques in the physical examination: Better at the bedside. Heart.

[14] Andersen G.N., Haugen B.O., Graven T., Salvesen O., Mjølstad O.C., Dalen H. (2011). Feasibility and reliability of point-of-care pocket-sized echocardiography. Eur. J. Echocardiogr..

[15] Michalski B., Kasprzak J.D., Szymczyk E., Lipiec P. (2012). Diagnostic utility and clinical usefulness of the pocket echocardiographic device. Echocardiography.

[16] Razi R., Estrada J.R., Doll J., Spencer K.T. (2011). Bedside hand carried ultrasound by internal medicine residents versus traditional clinical assessment for the identification of systolic dysfunction in patients admitted with decompensated heart failure. J. Am. Soc. Echocardiogr..

[17] Pastore M.C., Mandoli G.E., Aboumarie H.S., Santoro C., Bandera F., D’Andrea A., Benfari G., Esposito R., Evola V., Sorrentino R. (2020). Basic and advanced echocardiography in advanced heart failure: An overview. Heart Fail. Rev..

[18] Saeed S., Mohamed Ali A., Wasim D., Risnes I., Urheim S. (2023). Correlation between Murmurs and Echocardiographic Findings; From an Imaging Cardiologist Point of View. Curr. Probl. Cardiol..

[19] Vilacosta I., Olmos C., de Agustín A., López J., Islas F., Sarriá C., Ferrera C., Ortiz-Bautista C., Sánchez-Enrique C., Vivas D. (2015). The diagnostic ability of echocardiography for infective endocarditis and its associated complications. Expert Rev. Cardiovasc. Ther..

[20] Kuo D.C., Peacock W.F. (2015). Diagnosing and managing acute heart failure in the emergency department. Clin. Exp. Emerg. Med..

[21] McDonagh T.A., Metra M., Adamo M., Gardner R.S., Baumbach A., Böhm M., Burri H., Butler J., Čelutkienė J., Chioncel O. (2021). 2021 ESC Guidelines for the diagnosis and treatment of acute and chronic heart failure. Eur. Heart J..

[22] Dini F.L., Cameli M., Stefanini A., Aboumarie H.S., Lisi M., Lindqvist P., Henein M.Y. (2024). Echocardiography in the Assessment of Heart Failure Patients. Diagnostics.

[23] Neskovic A.N., Skinner H., Price S., Via G., De Hert S., Stankovic I., Galderisi M., Donal E., Muraru D., Sloth E. (2018). Reviewers: This document was reviewed by members of the 2016–2018 EACVI Scientific Documents Committee. Focus cardiac ultrasound core curriculum and core syllabus of the European Association of Cardiovascular Imaging. Eur. Heart J. Cardiovasc. Imaging.

[24] Neskovic A.N., Edvardsen T., Galderisi M., Garbi M., Gullace G., Jurcut R., Dalen H., Hagendorff A., Lancellotti P., European Association of Cardiovascular Imaging Document Reviewers (2014). Focus cardiac ultrasound: The European Association of Cardiovascular Imaging viewpoint. Eur. Heart J. Cardiovasc. Imaging.

[25] Galderisi M., Cosyns B., Edvardsen T., Cardim N., Delgado V., Di Salvo G., Donal E., Sade L.E., Ernande L., Garbi M. (2017). Standardization of adult transthoracic echocardiography reporting in agreement with recent chamber quantification, diastolic function, and heart valve disease recommendations: An expert consensus document of the European Association of Cardiovascular Imaging. Eur. Heart J. Cardiovasc. Imaging.

[26] Ruge M., Marhefka G.D. (2022). IVC measurement for the noninvasive evaluation of central venous pressure. J. Echocardiogr..

[27] Rudski L.G., Lai W.W., Afilalo J., Hua L., Handschumacher M.D., Chandrasekaran K., Solomon S.D., Louie E.K., Schiller N.B. (2010). Guidelines for the echocardiographic assessment of the right heart in adults: A report from the American Society of Echocardiography endorsed by the European Association of Echocardiography, a registered branch of the European Society of Cardiology, and the Canadian Society of Echocardiography. J. Am. Soc. Echocardiogr..

[28] Gargani L., Girerd N., Platz E., Pellicori P., Stankovic I., Palazzuoli A., Pivetta E., Miglioranza M.H., Soliman-Aboumarie H., Agricola E. (2023). This document was reviewed by members of the 2020–2022 EACVI Scientific Documents Committee. Lung ultrasound in acute and chronic heart failure: A clinical consensus statement of the European Association of Cardiovascular Imaging (EACVI). Eur. Heart J. Cardiovasc. Imaging.

[29] Lang R.M., Badano L.P., Mor-Avi V., Afilalo J., Armstrong A., Ernande L., Flachskampf F.A., Foster E., Goldstein S.A., Kuznetsova T. (2015). Recommendations for cardiac chamber quantification by echocardiography in adults: An update from the American Society of Echocardiography and the European Association of Cardiovascular Imaging. J. Am. Soc. Echocardiogr..

[30] Lichtenstein D.A. (2014). Lung ultrasound in the critically ill. Ann. Intensive Care.

[31] Lichtenstein D.A., Mezière G.A. (2008). Relevance of lung ultrasound in the diagnosis of acute respiratory failure: The BLUE protocol. Chest.

[32] Volpicelli G., Elbarbary M., Blaivas M., Lichtenstein D.A., Mathis G., Kirkpatrick A.W., Melniker L., Gargani L., Noble V.E., Via G. (2012). International Liaison Committee on Lung Ultrasound (ILC-LUS) for International Consensus Conference on Lung Ultrasound (ICC-LUS). International evidence-based recommendations for point-of-care lung ultrasound. Intensive Care Med..

[33] Parkin W.G., Leaning M.S. (2008). Therapeutic control of the circulation. J. Clin. Monit. Comput..

[34] Begic E., Begic Z. (2017). Accidental Heart Murmurs. Med. Arch..

[35] Shellenberger R.A., Crass S., Jevicks J., Badhwar A., Albright J., Kumar A. (2023). Bedside Physical Examination for the Diagnosis of Aortic Stenosis: A Systematic Review and Meta-analysis. CJC Open.

[36] Wen S., Naqvi T.Z. (2023). Point-of-Care Ultrasound in Detection, Severity and Mechanism of Significant Valvular Heart Disease and Clinical Management. J. Clin. Med..

[37] Vahanian A., Beyersdorf F., Praz F., Milojevic M., Baldus S., Bauersachs J., Capodanno D., Conradi L., De Bonis M., De Paulis R. (2022). 2021 ESC/EACTS Guidelines for the management of valvular heart disease. Eur. Heart J..

[38] Bai A.D., Steinberg M., Showler A., Burry L., Bhatia R.S., Tomlinson G.A., Bell C.M., Morris A.M. (2017). Diagnostic Accuracy of Transthoracic Echocardiography for Infective Endocarditis Findings Using Transesophageal Echocardiography as the Reference Standard: A Meta-Analysis. J. Am. Soc. Echocardiogr..

[39] Li M., Kim J.B., Sastry B.K.S., Chen M. (2024). Infective endocarditis. Lancet.

[40] Habib G., Badano L., Tribouilloy C., Vilacosta I., Zamorano J.L., Galderisi M., Voigt J.U., Sicari R., Cosyns B., Fox K. (2010). Recommendations for the practice of echocardiography in infective endocarditis. Eur. J. Echocardiogr..

[41] Horgan S.J., Mediratta A., Gillam L.D. (2020). Cardiovascular Imaging in Infective Endocarditis: A Multimodality Approach. Circ. Cardiovasc. Imaging.

[42] Tayal V.S., Kline J.A. (2003). Emergency echocardiography to detect pericardial effusion in patients in PEA and near-PEA states. Resuscitation.

[43] Haji D.L., Royse A., Royse C.F. (2013). Review article: Clinical impact of non-cardiologist-performed transthoracic echocardiography in emergency medicine, intensive care medicine and anaesthesia. Emerg. Med. Australas..

[44] Zanza C., Longhitano Y., Artico M., Cammarota G., Barbanera A., Racca F., Audo A., Ravera E., Migneco A., Piccioni A. (2020). Bedside Cardiac Pocus in Emergency Setting: A Practice Review. Rev. Recent Clin. Trials.

[45] Flamanc T., de Carvalho H., Le Bastard Q., Javaudin F., Pes P., Montassier E., Le Conte P. (2024). Impact of an enhanced focused cardiac ultrasound on treatment changes in a population of internal medicine patients. J. Clin. Ultrasound..

[46] Omar A.M., Bansal M., Sengupta P.P. (2016). Advances in Echocardiographic Imaging in Heart Failure with Reduced and Preserved Ejection Fraction. Circ. Res..

[47] Keikha M., Salehi-Marzijarani M., Soldoozi Nejat R., Sheikh Motahar Vahedi H., Mirrezaie S.M. (2018). Diagnostic Accuracy of Rapid Ultrasound in Shock (RUSH) Exam; A Systematic Review and Meta-analysis. Bull. Emerg. Trauma.

[48] Oh J.K., Park J.H. (2023). Role of echocardiography in acute pulmonary embolism. Korean J. Intern. Med..

[49] Pastore M.C., Ilardi F., Stefanini A., Mandoli G.E., Palermi S., Bandera F., Benfari G., Esposito R., Lisi M., Pasquini A. (2022). Bedside Ultrasound for Hemodynamic Monitoring in Cardiac Intensive Care Unit. J. Clin. Med..

[50] McConnell M.V., Solomon S.D., Rayan M.E., Come P.C., Goldhaber S.Z., Lee R.T. (1996). Regional right ventricular dysfunction detected by echocardiography in acute pulmonary embolism. Am. J. Cardiol..

[51] Konstantinides S.V., Meyer G., Becattini C., Bueno H., Geersing G.J., Harjola V.P., Huisman M.V., Humbert M., Jennings C.S., Jiménez D. (2020). 2019 ESC Guidelines for the diagnosis and management of acute pulmonary embolism developed in collaboration with the European Respiratory Society (ERS). Eur. Heart J..

[52] Kennedy Hall M., Coffey E.C., Herbst M., Liu R., Pare J.R., Andrew Taylor R., Thomas S., Moore C.L. (2015). The “5Es” of emergency physician-performed focused cardiac ultrasound: A protocol for rapid identification of effusion, ejection, equality, exit, and entrance. Acad. Emerg. Med..

[53] Imazio M., Mayosi B.M., Brucato A., Adler Y. (2010). Pericardial effusion triage. Int. J. Cardiol..

[54] Koh N., Nieman K. (2023). Role of cardiac imaging in acute chest pain. Br. J. Radiol..

[55] Liu F., Huang L. (2018). Usefulness of ultrasound in the management of aortic dissection. Rev. Cardiovasc. Med..

[56] Evangelista A., Flachskampf F.A., Erbel R., Antonini-Canterin F., Vlachopoulos C., Rocchi G., Sicari R., Nihoyannopoulos P., Zamorano J., European Association of Echocardiography (2010). Echocardiography in aortic diseases: EAE recommendations for clinical practice. Eur. J. Echocardiogr..

[57] Lancellotti P., Price S., Edvardsen T., Cosyns B., Neskovic A.N., Dulgheru R., Flachskampf F.A., Hassager C., Pasquet A., Gargani L. (2015). The use of echocardiography in acute cardiovascular care: Recommendations of the European Association of Cardiovascular Imaging and the Acute Cardiovascular Care Association. Eur. Heart J. Acute Cardiovasc. Care.

[58] Mathews B.K., Zwank M. (2017). Hospital Medicine Point of Care Ultrasound Credentialing: An Example Protocol. J. Hosp. Med..

[59] Dayan R.R., Karni O., Shitrit I.B., Gaufberg R., Ilan K., Fuchs L. (2025). Principles for Developing a Large-Scale Point-of-Care Ultrasound Education Program: Insights from a Tertiary University Medical Center in Israel. Perspect. Med. Educ..

[60] Elhassan M.G., Grewal S., Nezarat N. (2023). Point-of-Care Ultrasonography in Internal Medicine: Limitations and Pitfalls for Novice Users. Cureus.

[61] Jenkins S., Alabed S., Swift A., Marques G., Ryding A., Sawh C., Wardley J., Shah B.N., Swoboda P., Senior R. (2021). Diagnostic accuracy of handheld cardiac ultrasound device for assessment of left ventricular structure and function: Systematic review and meta-analysis. Heart.

[62] Perez-Sanchez A., Johnson G., Pucks N., Soni R.N., Lund T.J.S., Andrade A.J., Le M.T., Solis-McCarthy J., Wong T., Ashraf A. (2024). Comparison of 6 handheld ultrasound devices by point-of-care ultrasound experts: A cross-sectional study. Ultrasound J..

